# Cardiac Autonomic Modulation and Cognitive Performance in Community-Dwelling Older Adults: A Preliminary Study

**DOI:** 10.3390/neurolint17050074

**Published:** 2025-05-12

**Authors:** Paula Andreatta Maduro, Luiz Alcides Ramires Maduro, Polyana Evangelista Lima, Ana Clara Castro Silva, Rita de Cássia Montenegro da Silva, Alaine Souza Lima Rocha, Maria Jacqueline Silva Ribeiro, Juliana Magalhães Duarte Matoso, Bruno Bavaresco Gambassi, Paulo Adriano Schwingel

**Affiliations:** 1 Programa de Pós-Graduação em Ciências da Saúde (PPGCS), Universidade de Pernambuco (UPE), Recife 50100-130, PE, Brazil; pmaduro1@gmail.com (P.A.M.); polyana.uefs@gmail.com (P.E.L.); alaine.rocha@ufc.br (A.S.L.R.); 2 Laboratório de Pesquisas em Desempenho Humano (LAPEDH), Universidade de Pernambuco (UPE), Petrolina 56328-900, PE, Brazil; luiz.maduro@univasf.edu.br (L.A.R.M.); anaclara.silva@discente.univasf.edu.br (A.C.C.S.); rita.montenegrorrrb@gmail.com (R.d.C.M.d.S.); jacqueribeiro.cardio@uol.com.br (M.J.S.R.); juduartte@yahoo.com.br (J.M.D.M.); professorbrunobavaresco@gmail.com (B.B.G.); 3 Hospital de Ensino Dr. Washington Antônio de Barros da Universidade Federal do Vale do São Francisco (HU-UNIVASF), Empresa Brasileira de Serviços Hospitalares (EBSERH), Petrolina 56304-205, PE, Brazil; 4 Colegiado de Educação Física (CEFIS), Universidade Federal do Vale do São Francisco (UNIVASF), Petrolina 56304-917, PE, Brazil; 5 Programa de Pós-Graduação em Gestão e Atenção à Saúde (PPGGAS), Universidade Ceuma (UNICEUMA), São Luís 65075-120, MA, Brazil; 6 Departamento de Fisioterapia, Universidade Federal do Ceará (UFC), Fortaleza 60440-900, CE, Brazil; 7 Curso de Medicina, Centro de Ciências da Saúde (CCS), Universidade Estadual do Maranhão (UEMA), São Luís 65055-310, MA, Brazil; 8 Grupo de Pesquisa Clínica de Hipertensão Arterial e Doenças Metabólicas Associadas, Universidade do Estado do Rio de Janeiro (UERJ), Rio de Janeiro 20550-900, RJ, Brazil

**Keywords:** heart rate variability, autonomic nervous system, ageing, executive function, information processing speed, parasympathetic modulation

## Abstract

Background/Objectives: Cognitive decline has been increasingly linked to cardiac autonomic regulation; however, its specific associations with cognitive domains, such as information processing speed and executive function, remain unclear. This preliminary study examined the relationship between cardiac autonomic modulation and cognitive performance in older adults. Methods: A cross-sectional study was conducted with 101 older adults (aged ≥60 years) attending a university hospital outpatient clinic. Participants were classified as without cognitive impairment (WCI) or cognitively impaired and not demented (CIND) based on neuropsychological assessments. Heart rate variability (HRV) was measured at rest, focusing on the time-domain parameters (SDNN, rMSSD, and pNN50). Trail making test parts A and B (TMT-A and TMT-B) were used to assess information processing speed and executive function, respectively. Analyses of covariance (ANCOVAs) were performed, adjusting for confounding variables including age, sex, and comorbidities. Results: Participants in the CIND group had significantly lower HRV indices than those in the WCI group (SDNN, *p* < 0.05, *d* = 0.44; rMSSD, *p* < 0.05, *d* = 0.39; pNN50, *p* < 0.05, *d* = 0.40), indicating reduced parasympathetic modulation. Higher HRV values were observed in individuals with preserved processing speed and executive function. Specifically, pNN50 was significantly associated with processing speed (*p* = 0.04), and SDNN was significantly correlated with executive function (*p* = 0.02). These associations persisted even after adjusting for confounding factors. Conclusions: Reduced cardiac autonomic modulation, especially lower parasympathetic activity, is significantly associated with cognitive impairment in older adults. Lower pNN50 values were correlated with slower information processing speed, and lower SDNN was associated with poorer executive function. These findings support the potential use of HRV as a physiological biomarker to detect cognitive changes during ageing.

## 1. Introduction

Cognitive decline is a significant concern among older adults, impacting their independence and quality of life and increasing the risk of dementia and associated comorbidities [1,2]. The mechanisms underlying cognitive impairment are complex and involve neurodegenerative processes, vascular dysfunction, and metabolic disturbances [3]. Recent studies indicate that dysfunction of the autonomic nervous system (ANS), particularly a reduction in heart rate variability (HRV), may critically influence cognitive ageing trajectories [4,5]. For instance, research on populations with metabolic disorders such as type 2 diabetes mellitus (T2DM) has shown that reduced HRV correlates with delayed cognitive event-related potentials and lower cognitive screening scores, highlighting early interactions between autonomic function and mental health [6].

HRV, a well-established marker of cardiac autonomic regulation, reflects the balance between sympathetic and parasympathetic activity and has been linked to brain health and neurodegenerative processes [7]. Age-related autonomic decline, including decreased HRV, is associated with various adverse outcomes, including cardiovascular and metabolic dysfunction, impaired cognitive performance, and diminished functional independence [8]. These associations may be partly explained by the neurovisceral integration model, which suggests that cognitive, emotional, and autonomic processes share common neural pathways, particularly within the prefrontal cortex [9].

Previous research has identified links between reduced HRV and cognitive impairment, such as mild cognitive impairment (MCI) and Alzheimer’s disease [10,11]. Reduced parasympathetic activity, indicated by a lower root mean square of successive R–R interval differences (rMSSD) and a decreased percentage of successive R–R intervals differing by more than 50 ms (pNN50), has consistently been observed in individuals with cognitive dysfunction [12]. Nevertheless, the relationship between HRV and specific cognitive domains, such as information processing speed and executive function, remains relatively unexplored, particularly in older populations [13].

Information processing speed and executive function are critical cognitive domains that commonly decline with age and often serve as early indicators of neurodegenerative diseases [14]. Processing speed underlies the efficiency of mental operations and supports more complex cognitive tasks, whereas executive function governs decision-making, cognitive flexibility, and goal-directed behaviour [15]. Impairments in these domains are linked to an increased risk of cognitive dysfunction and a reduced quality of life among older adults [16]. The physiological mechanisms underlying these cognitive declines are not fully understood, despite their clinical importance. Examining autonomic control through HRV may provide valuable insights into the early neurophysiological dysfunction that precedes more severe cognitive impairment. Understanding these associations could inform preventive measures and targeted interventions for ageing populations.

Clarifying the relationship between HRV and cognitive decline may thus help identify early markers of neurodegeneration and guide preventive and therapeutic strategies [17]. Considering the rising global burden of cognitive disorders, it is essential to investigate modifiable physiological correlates such as HRV. This preliminary study aimed to examine the association between HRV and cognitive performance in older adults, focusing specifically on information processing speed and executive function. Its objectives were to compare HRV parameters between older adults with and without cognitive impairment, assess the relationships between HRV indices and performance in these cognitive domains, and explore whether changes in HRV are associated with early indicators of cognitive decline [18]. This study contributes to the growing body of literature on cardiovascular autonomic function and neurocognitive ageing, potentially supporting strategies to mitigate cognitive deterioration.

## 2. Materials and Methods

### 2.1. Study Design and Participants

This cross-sectional, descriptive, and analytical study was conducted following the translated [19] and updated version of the strengthening the reporting of observational studies in epidemiology (STROBE) statement [20], alongside the STROBE guidelines for reporting observational research [21].

The research was conducted at the outpatient clinic of the Dr Washington Antônio de Barros Teaching Hospital (HU), operated by the Brazilian Hospital Services Company (EBSERH) and affiliated with the Federal University of Vale do São Francisco (UNIVASF), located in Petrolina, Brazil. The study followed the ethical principles of the Declaration of Helsinki (1964, revised in 2013) and complied with the regulations established in Resolutions 466/2012 and 510/2016 of the Brazilian National Health Council. Ethical approval was obtained from the Research Ethics Committee of the School of Medical Sciences of Pernambuco (CEP-FCM/PE) under evaluation report No. 4.389.686 and certificate of presentation for ethical appraisal (CAAE) No. 38942320.4.0000.5192. All participants provided written informed consent by signing the informed consent form (ICF) before enrolment.

A total of 105 participants aged 60 years or older were recruited. The sample size was calculated based on a total elderly population of 20,733 residents in Petrolina, considering a maximum expected proportion of 50%, a margin of error of 5%, an anticipated loss of 20%, and a 95% confidence level [22].

Inclusion criteria were age ≥ 60 years, residence in Petrolina, at least four years of education, and either sex, with no income restrictions. Exclusion criteria included the following: (a) Beck depression inventory (BDI) score > 19 [23]; (b) uncorrected motor or sensory deficits affecting neuropsychological assessment; (c) recent medication changes (within four weeks); (d) use of psychotropic drugs or beta-blockers; (e) use of ≥4 antihypertensive medications; (f) systolic blood pressure (SBP) ≥ 180 mmHg or diastolic blood pressure (DBP) ≥ 110 mmHg; (g) history of significant cardiovascular events or interventions; (h) Parkinson’s disease diagnosis; (i) history of stroke or transient ischemic attack; and (j) untreated hypothyroidism.

**Figure 1:**
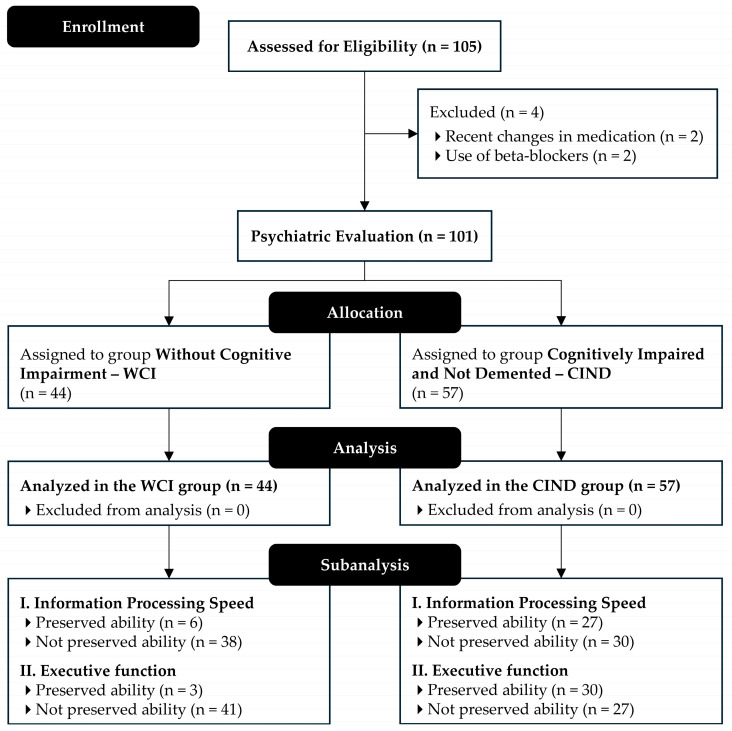
Flow diagram of data collection and analysis procedures.

Eligible participants were screened using the mini-mental state examination (MMSE), which was adjusted for education [24]. Those identified with potential cognitive impairment underwent psychiatric evaluation and were classified as either without cognitive impairment (WCI) or cognitively impaired and not demented (CIND). Figure 1 illustrates participant eligibility.

### 2.2. Procedures

Participants were recruited through public announcements at HU-UNIVASF and on social media. Interested individuals received informational letters and the ICF. Upon consent, assessment sessions were scheduled at the HU-UNIVASF outpatient clinic.

Assessments occurred in a quiet room and were conducted exclusively in the morning to minimise circadian effects. Initially, participants completed structured interviews to gather sociodemographic data and health history. Cognitive function was assessed using the MMSE. Participants with signs of impairment underwent psychiatric evaluation before a second visit.

On the second visit, after resting supine for 10 min, participants underwent haemodynamic and cardiac autonomic evaluations, followed by anthropometric measurements using International Society for the Advancement of Kinanthropometry (ISAK) standards [25]. Functional capacity assessments occurred on the third visit. All procedures followed standardised protocols conducted by trained professionals.

All assessments were carried out in strict compliance with standardised protocols to ensure methodological rigour and data reliability. Trained professionals conducted all procedures under controlled conditions, thereby ensuring consistency and minimising the risk of bias.

### 2.3. Haemodynamic Parameters and Cardiac Autonomic Function Assessments

Arterial blood pressure (BP) was measured following the recommendations outlined in the Brazilian Hypertension guidelines [26] and the updated Brazilian guidelines for In-Office and Out-of-Office BP Measurement [27]. Three consecutive readings were obtained using an automated device (HEM-7130, OMRON Healthcare, Inc., Lake Forest, CA, USA) following a 10-minute rest period, in line with current best practice standards for accurate BP assessment. Measurements were taken on the non-dominant arm, with cuff sizes selected according to the participant’s arm circumference. The mean of the final two readings was used as the representative BP value.

HRV was assessed using R–R interval recordings obtained via a free smartphone application (Elite HRV LLC, Asheville, NC, USA, release 4.0.2, 2018) on Android, connected via Bluetooth 4.0 to a Polar H10 wireless chest strap transmitter (Polar Electro Oy, Kempele, Finland) [28]. This system has been validated against a gold-standard electrocardiogram (ECG) in asymptomatic adults, demonstrating excellent agreement for R–R interval recordings at rest (*r* = 0.999) and during orthostatic challenge (*r* = 0.988), with minimal bias and narrow limits of agreement [28]. No significant differences were found between methods for key HRV indices, including SDNN, rMSSD, pNN50, LF, and HF [28]. Although smartphone-based HRV measurements are not entirely equivalent to standard ECG-based assessments, this approach offers a practical and validated method for non-invasive monitoring under controlled resting conditions. Finally, the signals were transmitted to a computer for further analysis.

Before data collection, participants were instructed to abstain from consuming alcoholic and stimulant beverages (e.g., soft drinks, coffee, chocolate milk, green tea) and from engaging in strenuous physical activity on the day of and the day before the assessment [29]. Additionally, participants were asked to avoid speaking, moving, coughing, or falling asleep during the measurement. All participants rested in the supine position for at least 10 min before data acquisition commenced. HRV was then recorded for 10 min under spontaneous breathing conditions while participants remained at rest. Short-term recordings of this duration have been shown to provide HRV values that are comparable to those obtained through long-term monitoring and are frequently used due to their practicality.

All HRV measurements were collected in the morning. The R–R interval data were exported to the Kubios HRV software (Kubios Oy, Kuopio, Finland, version 2.2) for analysis using time-domain and frequency-domain methodologies. The following time-domain parameters were computed: mean R–R interval, standard deviation of normal-to-normal intervals (SDNN), rMSSD, and pNN50. In the frequency domain, spectral power was assessed within the physiologically relevant range of 0.04 to 0.4 Hz. The low-frequency (LF; 0.04–0.15 Hz) and high-frequency (HF; 0.15–0.4 Hz) components were calculated in both absolute units (ms^2^) and normalised units (nu). The LF and HF bands were interpreted as indices of sympathetic and parasympathetic autonomic modulation, respectively. The LF/HF ratio was used as an indicator of cardiac sympathovagal balance [30].

### 2.4. Anthropometric Measurements

Total body mass (TBM), measured in kilograms (kg), and height, measured in centimetres (cm), were assessed using a calibrated anthropometric scale (PL-200, Filizola S.A. Pesagem e Automação, São Paulo, SP, Brazil), certified under NBR ISO/IEC 17025:2005, with a precision of 0.05 kg and 0.1 cm. Body mass index (BMI, kg/m^2^) was subsequently calculated by dividing the TBM by the square of the individual’s height in metres (m^2^).

### 2.5. Autonomic Nervous System Dysfunction

Assessment of ANS dysfunction was carried out by evaluating neurogenic orthostatic hypotension (OH), as defined by the International Consensus Statement [31]. Neurogenic OH was assessed through BP measurements taken in both supine and standing positions using an automated device (HEM-7130, OMRON Healthcare, Inc.).

The supine BP was calculated as the average of the third measurement obtained following a thirty-minute rest period in the supine position. Participants were then instructed to stand unassisted, and BP measurements were taken at the first, second, and third minutes of active standing. Throughout these assessments, participants were asked to remain relaxed and to avoid unnecessary movements or muscle contractions that could affect BP regulation. Neurogenic OH was defined as a drop in SBP of at least 20 mmHg and/or a drop in DBP of at least 10 mmHg occurring within three minutes of standing.

### 2.6. Assessment of Activities of Daily Living and Instrumental Activities of Daily Living

Functional assessment was conducted using the Katz index of independence in activities of daily living [32] and the Lawton instrumental activities of daily living scale [33], with the latter adapted for use in the Brazilian older adult population [34].

The Katz index evaluates an individual’s level of independence in performing basic activities of daily living (ADLs). It assesses six self-care tasks, arranged hierarchically by complexity: bathing, dressing, toileting, transferring, continence, and feeding. Each task is scored dichotomously—independent (1 point) or dependent (0 points)—yielding a total score ranging from 0 to 6 [32]. A score of 6 indicates full independence, whereas a score of 0 reflects total dependence across all assessed activities.

By contrast, the Lawton scale evaluates an individual’s ability to perform instrumental activities of daily living (IADLs), which require greater cognitive and physical capacity [33]. The version used in this study was the Brazilian adaptation [34], comprising nine items that assess competencies such as telephone use, shopping, food preparation, housekeeping, laundry, transportation, medication management, financial handling, and minor household repairs. Scores range from 9 to 27, with higher scores indicating greater functional independence. Participants scoring ≤ 14 were classified as dependent for IADLs.

### 2.7. Assessments of Information Processing Speed and Executive Function

To evaluate executive function and information processing speed, the trail making test (TMT) parts A and B were administered [35,36]. Part A assessed information processing speed, whilst part B targeted executive function, with particular emphasis on cognitive flexibility.

Each part consists of 25 circles dispersed across a sheet of paper. In part A, the circles are numbered sequentially from 1 to 25, and participants are instructed to connect the numbers in ascending order. In part B, the circles contain both numbers (1 to 13) and letters (A to L); participants must connect them in ascending order while alternating between numbers and letters (i.e., 1 → A → 2 → B → 3 → C, and so on).

Participants were instructed to complete the task as quickly as possible without lifting the pen or pencil. Any errors were immediately identified by the evaluator, who prompted the participant to correct them. The time required for correction was included in the overall task completion time. The test was concluded when the participant either completed the sequence or chose to discontinue [36].

Performance classification was based on normative values proposed by Ashendorf et al. [35]. For TMT part A, the cut-off time indicating preserved ability was ≤42 s for individuals aged 60–74 years and ≤51 s for those aged 75 or older. For TMT part B, the respective cut-offs were 101 s and 128 s. Participants exceeding these thresholds were classified as having no preserved ability in the respective domain.

### 2.8. Statistical Analysis

Data were double-entered and analysed using the Statistical Package for the Social Sciences (SPSS Inc., Chicago, IL, USA, release 16.0.2, 2007). The normality of continuous variables was assessed using the Shapiro–Wilk test, while Levene’s test was employed to examine the homogeneity of variances. Categorical variables were presented as absolute (*n*) and relative (%) frequencies. As most continuous variables did not meet assumptions of normality, they were reported as medians with corresponding interquartile ranges (first quartile–third quartile).

To compare outcomes between the WCI and CIND groups, the Mann–Whitney *U* test was applied for non-parametric variables, whereas the independent *t*-test was used where parametric assumptions were satisfied. Additionally, differences in HRV parameters among older adults with and without preserved cognitive function (based on trial making test performance thresholds) were analysed using the Kruskal–Wallis test, followed by Dunn’s post hoc test where appropriate.

Effect sizes (ESs) were calculated to quantify the magnitude of between-group differences, following the recommendations of Erceg-Hurn and Mirosevich [37] and Grissom [38]. To control for potential confounding variables—namely, age, years of education, and the presence of systemic arterial hypertension (SAH)—a separate analysis of covariance (ANCOVA) was performed for each HRV parameter. Adjusted means were computed for each group and differences were assessed using *F*-tests. Partial eta squared (η^2^_p_) was reported as a measure of effect size.

All *p*-values and 95% confidence intervals (95% CIs) were calculated and reported with exact values. A two-tailed significance level of 5% (*p* ≤ 0.05) was adopted for all statistical tests.

## 3. Results

### 3.1. Sample Characteristics

A total of 101 older adults were included in the analysis. Participants in the cognitively impaired and not demented (CIND) group were significantly older (*p* < 0.001), had fewer years of education (*p* = 0.001), and scored lower on the mini-mental state examination (MMSE; *p* < 0.001) compared to the without cognitive impairment (WCI) group. Additionally, the CIND group exhibited poorer performance in both basic activities of daily living (ADLs; *p* < 0.001) and instrumental activities of daily living (IADLs; *p* < 0.001). The CIND participants also demonstrated slower performance on tests measuring information processing speed (*p* < 0.001) and executive function (*p* < 0.001). No significant differences were noted in body mass index (BMI), alcohol or tobacco use, or hypertension prevalence between the groups (Table 1).

### 3.2. Haemodynamic Parameters

There were no significant differences between groups regarding neurogenic OH, resting heart rate, SBP, or DBP. The neurogenic OH was present in 39.6% of the total sample, distributed similarly across groups (WCI = 40.9% and CIND = 38.6%; *p* = 0.81). Resting heart rate (*p* = 0.38), SBP (*p* = 0.83), and DBP (*p* = 0.67) were also similar between groups (Table 2).

### 3.3. Heart Rate Variability

Significant differences in HRV time-domain parameters were found between groups, with the CIND group displaying lower values compared to the WCI group (Table 3). Specifically, SDNN (*p* = 0.035 and *d* = 0.44), rMSSD (*p* = 0.049 and *d* = 0.39), and pNN50 (*p* = 0.047 and *d* = 0.40) were significantly reduced in the CIND group, each demonstrating moderate effect sizes. No significant differences were observed for frequency-domain parameters (LF, HF, and LF/HF ratio).

To account for potential confounding variables, analyses of covariance (ANCOVAs) were conducted, adjusting for age, years of education, and the presence of SAH. Separate ANCOVA models were applied for time-domain and frequency-domain HRV indices. After adjustment, statistically significant group differences persisted for three time-domain measures. SDNN remained significantly lower in the CIND group (*p* = 0.035 and η^2^_p_ = 0.044; 95% CI: 0.001–0.127), consistent with a moderate effect size. Similarly, rMSSD was significantly reduced (*p* = 0.049 and η^2^_p_ = 0.039; 95% CI: 0.000–0.108), suggesting diminished parasympathetic modulation. A comparable reduction in pNN50 was also observed (*p* = 0.047 and η^2^_p_ = 0.040; 95% CI: 0.000–0.115), further supporting an association between vagal activity and cognitive function. Conversely, no significant group differences were identified for frequency-domain measures following adjustment. Specifically, LF (*p* = 0.796 and η^2^_p_ = 0.001; 95% CI: 0.000–0.010), HF (*p* = 0.751 and η^2^_p_ = 0.001; 95% CI: 0.000–0.011), and the LF/HF ratio (*p* = 0.509 and η^2^_p_ = 0.005; 95% CI: 0.000–0.012) all remained statistically non-significant. These findings indicate that reductions in time-domain HRV parameters are independently associated with cognitive impairment, whereas spectral indices of autonomic balance do not appear to discriminate between cognitively preserved and impaired individuals.

### 3.4. Heart Rate Variability and Cognitive Performance

**Figure 2:**
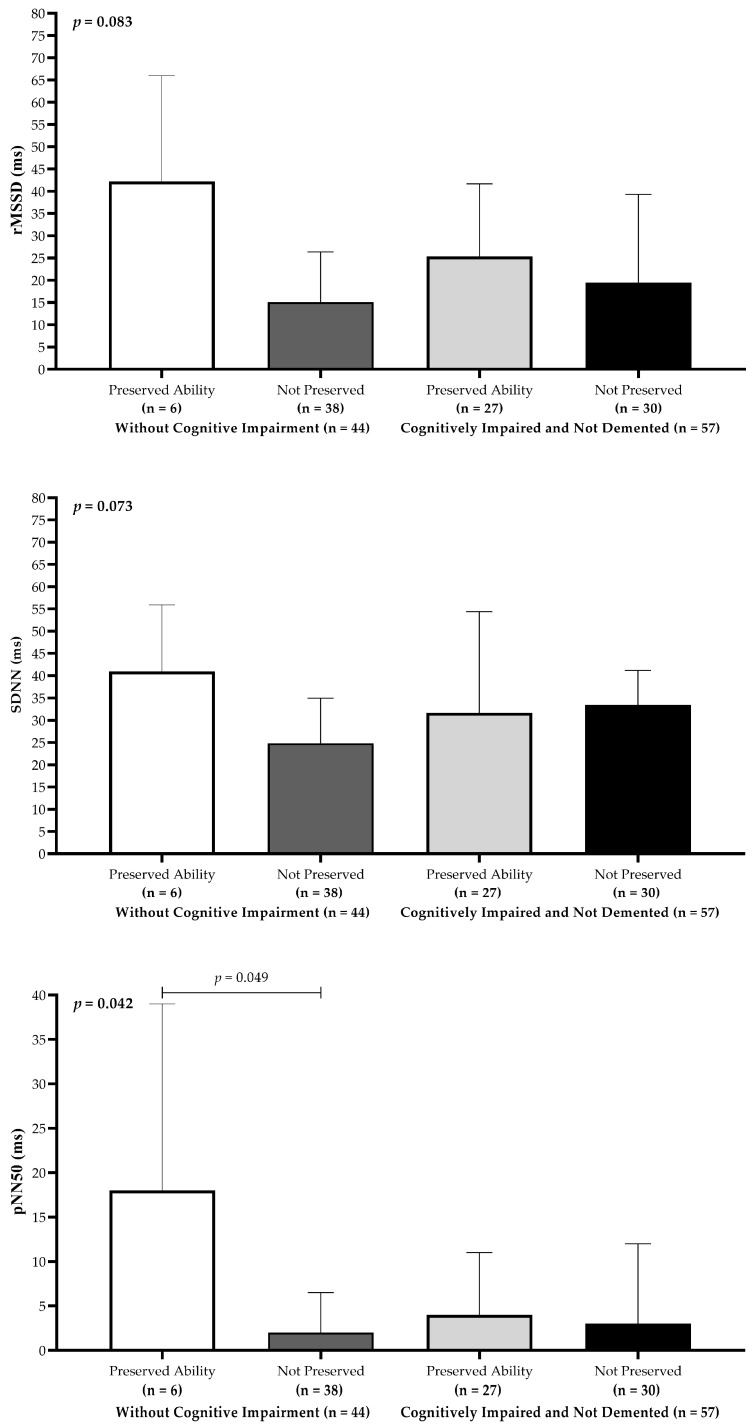
Heart rate variability time-domain parameters (rMSSD, SDNN, and pNN50) stratified by preserved versus impaired information processing speed in older adults.

Older adults classified in the WCI group with preserved information processing speed exhibited the highest median HRV values across all time-domain indices (Figure 2). Statistically significant differences were identified among the groups, with pNN50 showing the most pronounced difference (*p* < 0.05). Dunn’s post hoc analysis revealed that participants in the WCI group with preserved processing speed had significantly higher pNN50 values compared to those in the WCI group without preserved processing speed.

**Figure 3:**
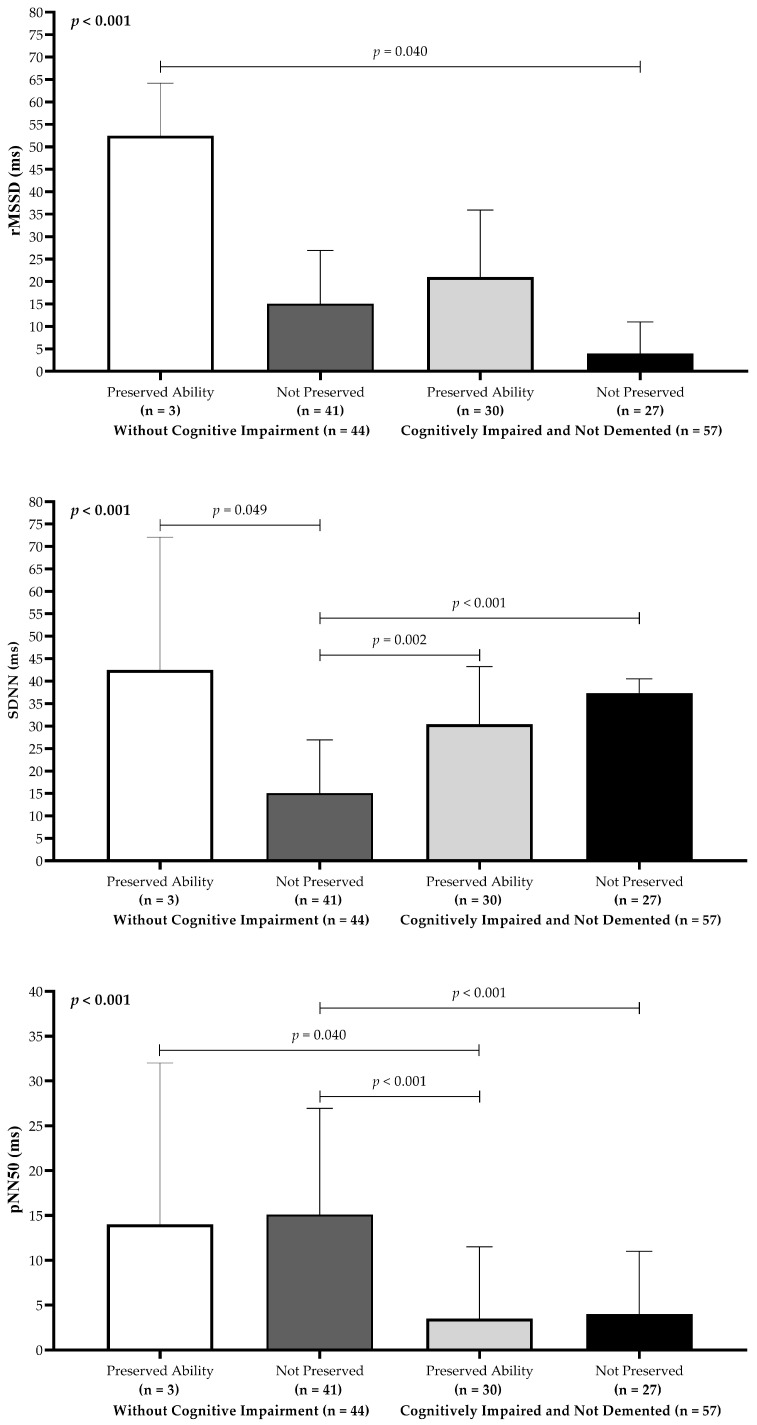
Heart rate variability time-domain parameters (rMSSD, SDNN, and pNN50) stratified by preserved versus impaired executive function in older adults.

A similar pattern emerged for executive function (Figure 3). Participants in the WCI group with preserved executive function displayed higher median HRV values across all time-domain metrics. Notably, rMSSD values were significantly higher (*p* < 0.05) in WCI participants with preserved executive function compared to CIND participants without preserved executive function. Additionally, WCI participants without preserved executive function exhibited significantly lower median SDNN values (*p* < 0.001) than all other comparison groups. Median pNN50 values were also significantly higher in WCI participants than in CIND participants, even among those with preserved executive function (*p* < 0.05). Finally, Dunn’s post hoc comparisons indicated that WCI participants without preserved executive function had significantly higher pNN50 scores compared to their CIND counterparts without preserved executive function.

## 4. Discussion

This preliminary study demonstrated an association between cognitive performance, particularly information processing speed, executive function, and cardiac autonomic regulation, as indicated by time-domain HRV parameters. Older adults with preserved cognitive function displayed significantly higher HRV values, especially rMSSD, SDNN, and pNN50, suggesting enhanced parasympathetic modulation. Conversely, participants with cognitive impairment showed substantially reduced HRV, underscoring the importance of autonomic function in cognitive health. These findings align with previous research connecting lower HRV to cognitive deficits [18,39,40] and highlight parasympathetic activity as essential for attention, memory, and inhibitory control. Consequently, HRV could serve as a physiological indicator of cognitive resilience during ageing [41,42].

Subgroup analyses reinforced these associations, revealing that participants with preserved processing speed had higher HRV values than those with impaired processing speed. Notably, pNN50 was particularly sensitive to differences in processing speed, supporting its role in rapid cognitive operations mediated by parasympathetic activity. Similarly, deficits in executive function were linked to reductions in SDNN and rMSSD, suggesting that autonomic flexibility is crucial for executive processes such as decision-making and attention regulation. These results are consistent with broader evidence linking autonomic modulation to cognitive adaptability [13].

Previous studies have frequently reported associations between cognitive impairment and reduced HRV, specifically in short-term measures that reflect vagal tone [12,43]. In line with this, our findings showed significant reductions in SDNN, rMSSD, and pNN50 in cognitively impaired individuals, reinforcing the sensitivity of these parameters to cognitive decline [44]. Reduced SDNN, reflecting overall autonomic regulation, may indicate diminished cardiovascular adaptability due to neurodegenerative changes that affect central autonomic control. This interpretation is supported by the neurovisceral integration model [45], which highlights shared neural substrates for cognitive and autonomic functions, particularly within prefrontal regions. Degeneration in these areas could simultaneously impair HRV and cognitive function, with cholinergic dysfunction playing a central role in both [4,17].

In contrast, the frequency-domain measures (LF, HF, and LF/HF ratio) did not differ significantly between the groups. Although some studies have linked cognitive impairment to altered spectral measures [12,42], our results suggest that these indices may be less sensitive to early cognitive changes than time-domain metrics. This difference might reflect the greater sensitivity of time-domain parameters to rapid fluctuations associated with cognitive tasks, whereas spectral indices may reflect more stable autonomic states [32,46]. This interpretation aligns with evidence from clinical populations, such as individuals with spinal cord injury, where time-domain HRV markers show limited sensitivity across subgroups and frequency-domain metrics (for example, LF/HF) demonstrate interpretive ambiguity at rest [47]. Methodological variations, including differences in measurement conditions and populations, may have contributed to discrepancies across studies [48].

We followed recent recommendations [49] emphasising the complexity of autonomic contributions to HRV. Frequency-domain metrics, such as LF and LF/HF ratio, which are traditionally viewed as sympathetic markers, also contain substantial parasympathetic influences at rest. Thus, we prioritised time-domain indices (SDNN, rMSSD, and pNN50) as more reliable markers of vagal tone, strengthening our conclusions regarding autonomic-cognitive associations.

Recently, alternative approaches to HRV assessment have emerged, including entropy-based methods derived from standard ECG recordings. In particular, the baroreflex entropy index (BEI) obtained from lead II ECG signals has demonstrated robust reproducibility and significant correlations with glycaemic markers in individuals with T2DM [49]. Unlike conventional frequency-domain HRV metrics, BEI leverages the complexity of R–R interval fluctuations and R-wave amplitudes to provide a refined analysis of autonomic function. While the BEI primarily focuses on assessing baroreflex sensitivity and biological system complexity, it further underscores the need for advanced HRV-derived markers capable of capturing subtle autonomic impairments. Although our study employed standard HRV indices without entropy-based analysis, future research integrating such advanced metrics could yield deeper insight into the interplay between autonomic regulation and cognitive decline.

Additionally, time-domain indices such as SDNN, rMSSD, and pNN50 may be more sensitive to early or subtle impairments in parasympathetic modulation, whereas alterations in spectral measures may only emerge with more advanced autonomic or cognitive decline. Discrepancies between studies could also stem from differences in HRV signal-processing techniques, measurement contexts (e.g., supine rest vs. active challenge), and cohort heterogeneity. Further longitudinal investigations combining time- and frequency-domain analyses are warranted to delineate the progression of autonomic dysfunction across different stages of cognitive ageing.

Several mechanisms may plausibly underlie these observed associations. Cholinergic dysfunction, frequently found in Alzheimer’s disease and related dementia, impairs both parasympathetic regulation and cognitive function [17,41]. Autonomic dysfunction has also been linked to vascular pathologies, such as arterial stiffness and endothelial dysfunction, which reduce cerebral perfusion and impair cognition [48]. Furthermore, chronic low-grade inflammation and oxidative stress may simultaneously disrupt autonomic regulation and neural integrity, reinforcing the bidirectional cycle of neurodegeneration [50]. Reduced HRV has also been associated with maladaptive stress responses, including hyperactivation of the hypothalamic–pituitary–adrenal (HPA) axis which is implicated in impairments in memory and executive function [13]. Evidence from populations with metabolic disorders, such as T2DM, supports this link, with studies reporting that HRV reductions correspond to delays in cognitive event-related potentials, such as P300 latency [6]. In addition, autonomic dysregulation in older adults has been shown to impair physiological reactivity and motivational processes, thereby reducing functional independence and increasing vulnerability to cognitive and behavioural decline [8].

Given its non-invasive nature and relative ease of implementation, HRV shows potential as an indicator for identifying associations with cognitive decline risk in older adults. This potential is further supported by studies indicating that HRV indices, such as SDNN and HF, are positively associated with health-related quality of life in cognitively impaired individuals [9]. The moderate effect sizes observed in the present study (e.g., Cohen’s *d* ≈ 0.39–0.44; η^2^_p_ ≈ 0.04) underscore the clinical relevance of these measures in distinguishing cognitively intact from impaired individuals. In future applications, time-domain HRV metrics, particularly pNN50, rMSSD, and SDNN, could be integrated into routine cognitive screening protocols to facilitate early detection of neurodegenerative risk. Moreover, HRV-based assessments may support the timely implementation of therapeutic strategies such as autonomic biofeedback, vagus nerve stimulation, or mindfulness-based interventions designed to enhance parasympathetic tone and prevent further cognitive deterioration.

Several limitations of this study should be considered when interpreting these findings. First, the cross-sectional design restricts causal inference, making it unclear whether reduced HRV precedes cognitive impairment or simply co-occurs as a manifestation of the underlying neurodegenerative processes. Second, the sample was composed of only community-dwelling older adults, which may limit the generalisability of the results to institutionalised populations or individuals with advanced cognitive decline. Although adjustments were made for key confounding variables such as age, education, and SAH, other potentially influential factors, including physical activity level, sleep quality, medication use, and comorbidities, were not directly assessed. These factors may influence both the cardiac autonomic regulation and cognitive performance. Their absence as covariates in the present analysis represents a limitation that could affect the interpretation of observed associations. Future research incorporating comprehensive assessments of these variables is warranted to enhance confounder control and to provide a more nuanced understanding of the interplay between autonomic and cognitive health in ageing populations. In addition, subgroup analyses were constrained by small sample sizes in certain strata, particularly among participants with preserved cognitive performance, thereby reducing the inferential strength of some comparisons. Future investigations involving larger, more heterogeneous cohorts are needed to validate these preliminary findings and elucidate the temporal and mechanistic relationships between autonomic regulation and cognitive function more comprehensively.

Moreover, the absence of data on inflammatory biomarkers (e.g., high-sensitivity C-reactive protein) and direct vascular assessments (e.g., shear stress or arterial stiffness) precluded the empirical evaluation of specific mechanistic pathways discussed in the literature. Although these mechanisms were considered within our theoretical framework, they could not be tested directly in this study. Future investigations integrating HRV metrics with biochemical and vascular biomarkers are essential to substantiate and expand on these hypotheses, thereby contributing to a more holistic understanding of the physiological underpinnings of cognitive ageing.

Furthermore, the exclusion of participants with untreated hypothyroidism, recent medication changes, or the use of psychotropic or beta-blocker medications was designed to minimise any potential confounding effects on HRV and cognitive performance. While these exclusions enhanced the internal validity of the study, they may have limited the generalisability of the findings to the broader older adult population, particularly individuals with more complex comorbidity profiles or pharmacological regimens. Subsequent studies should include more diverse samples to better characterise the impact of autonomic dysregulation in heterogeneous ageing populations. Additionally, although HRV measurements were conducted using a validated smartphone-based system (Elite HRV with Polar H10), it should be noted that this method is not identical to standard multilead ECG recordings. Although validation studies support its reliability under resting conditions [28], minor discrepancies cannot be excluded. Future studies employing standard ECG-based HRV analyses may enhance the precision and generalisability of these findings.

Collectively, these findings provide novel insights into the relationship between cardiac autonomic modulation and domain-specific cognitive performance in older adults. Unlike many previous studies that primarily examined global cognitive scores or dementia diagnosis, our study specifically focused on information processing speed and executive function domains recognised as early markers of neurodegeneration [51], using rigorous neuropsychological categorisation. By employing validated time-domain HRV parameters [52] and controlling for major confounders, such as age, education, and hypertension, we demonstrated that reductions in short-term parasympathetic modulation are associated with early-stage cognitive dysfunction. These results extend previous knowledge by supporting the use of time-domain HRV metrics as sensitive physiological correlates of cognitive ageing [52] and highlighting their potential application in preventive cognitive screening strategies [53].

Although our findings suggest that time-domain HRV indices may serve as accessible physiological correlates of early cognitive changes, caution must be exercised before advocating for their use in clinical screening. HRV parameters are influenced by a variety of factors, including physical fitness, mental health status, medication use, and comorbidities, which may limit their specificity for cognitive dysfunction [52]. Moreover, normative HRV values vary widely across populations and measurement protocols [52,54], complicating standardisation efforts. Consequently, while HRV holds promise as a complementary marker of cognitive health, further longitudinal studies are required to establish its predictive validity, clinical applicability, and integration into multimodal screening approaches.

Beyond its potential as a biomarker, autonomic modulation is a promising therapeutic target for cognitive health preservation. Non-invasive vagus nerve stimulation (nVNS) and HRV biofeedback (HRVB) interventions have been shown to enhance parasympathetic activity and improve autonomic regulation [55,56]. Preliminary studies suggest that nVNS may positively influence cognitive domains such as attention, memory, and executive function [55]. Similarly, HRVB has demonstrated potential benefits in working memory, processing speed, and stress resilience [56]. Autonomic modulation strategies, such as reducing systemic inflammation and supporting neural plasticity, have been implicated in broader neurophysiological mechanisms relevant to cognitive health, such as reducing systemic inflammation and supporting neural plasticity [57]. However, although these interventions are conceptually appealing, robust longitudinal trials specifically targeting cognitive outcomes in older adults remain limited. Future research is needed to confirm the clinical utility and long-term effects of autonomy-targeted therapies on cognitive ageing.

In summary, this preliminary study reinforces the notion that time-domain HRV metrics are reduced in older adults with cognitive impairment, reflecting diminished parasympathetic modulation and autonomic adaptability. The absence of significant differences in frequency-domain measures suggests that short-term fluctuations in HRV may capture early cognitive changes more reliably than spectral indices. Collectively, these findings indicate that HRV may serve as a clinically informative and accessible correlate of cognitive health in ageing populations, warranting further investigation through integrated approaches involving neuroimaging, biochemical profiling, vascular assessments, and longitudinal designs to fully elucidate the neurophysiological mechanisms underlying cognitive ageing.

## 5. Conclusions

This study highlights a significant relationship between cardiac autonomic regulation and cognitive performance in older adults. Participants with preserved processing speed and executive function showed higher values on time-domain HRV measures (SDNN, rMSSD, and pNN50), indicating enhanced parasympathetic activity. Conversely, cognitively impaired individuals exhibit lower HRV values, suggesting reduced autonomic adaptability.

Our findings particularly emphasise pNN50 as a sensitive marker for executive function, whereas SDNN is closely related to processing speed efficiency. The lack of significant differences in frequency-domain HRV parameters further underscores the relevance of short-term time-domain metrics for the early detection of cognitive changes.

Given the increasing recognition of autonomic–cognitive links, integrating HRV assessments into clinical practice could aid in the early identification of cognitive decline risk. Future studies employing longitudinal and multimodal approaches are recommended to clarify causal relationships and to explore whether interventions targeting autonomic function can enhance cognitive resilience in ageing populations.

## Figures and Tables

**Table 1:** Demographic, clinical, and cognitive characteristics of older adults with (CIND) and without cognitive impairment (WCI).

Variables	Total (*n* = 101)	WCI (*n* = 44)	CIND (*n* = 57)	*p*
	Mean ± SD	Mean ± SD	Mean ± SD	
Age, years	69.1 ± 6.6	66.2 ± 4.0	71.4 ± 7.4	<0.001
Female sex, *n* (%)	68 (67.3)	31 (70.5)	37 (64.9)	0.556
Schooling, years	8.3 ± 3.3	9.6 ± 3.1	7.4 ± 3.1	0.001
Mini-mental state examination, *n*	24.1 ± 4.0	26.6 ± 2.3	22.3 ± 4.1	<0.001
Total body mass, kg	70.6 ± 15.2	72.9 ± 15.9	68.8 ± 14.5	0.184
Height, cm	157.2 ± 7.6	159.9 ± 6.6	155.8 ± 8.0	0.033
Body mass index, kg/m^2^	28.5 ± 5.4	28.7 ± 5.6	28.3 ± 5.3	0.682
Obesity, *n* (%)	56 (55.4)	26 (59.1)	30 (52.6)	0.517
Alcohol use, *n* (%)	9 (8.9)	5 (11.4)	4 (7.0)	0.447
Tobacco, *n* (%)	6 (5.9)	1 (2.3)	5 (8.8)	0.171
Coronavirus infection, *n* (%)	23 (22.8)	13 (29.5)	10 (17.5)	0.154
Systemic arterial hypertension, *n* (%)	73 (72.3)	31 (70.5)	42 (73.7)	0.719
Activities of daily living (ADLs), *n*	5.1 ± 1.1	6.0 ± 0.1	4.3 ± 1.0	<0.001
Instrumental ADLs, *n*	23.5 ± 3.4	25.8 ± 0.8	21.7 ± 3.6	<0.001
Information processing speed, seconds	71.8 ± 47.6	49.7 ± 18.3	88.8 ± 55.7	<0.001
Executive function, seconds	225.1 ± 117.1	147.3 ± 63.2	285.2 ± 114.0	<0.001

WCI: without cognitive impairment; CIND: cognitively impaired and not demented.

**Table 2:** Haemodynamic parameters in older adults with (CIND) and without cognitive impairment (WCI).

Variables	Total (*n* = 101)	WCI (*n* = 44)	CIND (*n* = 57)	*p*	*d* (95% CI)
	Mean ± SD	Mean ± SD	Mean ± SD		
Resting heart rate, bpm	64.4 ± 11.1	65.5 ± 10.9	63.5 ± 11.3	0.378	0.18 (−0.22–0.57)
Systolic blood pressure, mmHg	115.1 ± 12.8	114.8 ± 14.2	115.3 ± 11.9	0.828	0.04 (−0.44–0.35)
Diastolic blood pressure, mmHg	73.8 ± 8.0	74.2 ± 8.3	73.5 ± 7.8	0.667	0.09 (−0.31–0.48)
Orthostatic hypotension, *n* (%)	40 (39.6)	18 (40.9)	22 (38.6)	0.814	0.03 (−0.43–0.34)

WCI: without cognitive impairment; CIND: cognitively impaired and not demented; SD: standard deviation; CI: confidence interval.

**Table 3:** Heart rate variability parameters in older adults with (CIND) and without cognitive impairment (WCI).

Variables	Total (*n* = 101)	WCI (*n* = 44)	CIND (*n* = 57)	*p*	*d* (95% CI)
	(1Q–3Q)	(1Q–3Q)	(1Q–3Q)		
Mean R–R interval, ms	849.3 (742.4–945.1)	856.4 (737.6–954.2)	833.0 (749.3–939.3)	0.795	0.05 (−0.32–0.46)
SDNN, ms	20.4 (12.0–37.0)	21.2 (14.6–41.5)	15.6 (9.3–28.9)	0.035	0.44 (0.04–0.83)
rMSSD, ms	30.7 (21.4–41.5)	32.6 (25.5–41.8)	26.2 (18.0–40.6)	0.049	0.39 (0.01–0.79)
pNN50, %	3.0 (2.0–9.5)	4.0 (2.5–10.0)	2.5 (2.0–9.5)	0.047	0.40 (0.01–0.80)
Low frequency (LF), ms^2^	106.0 (56.9–197.2)	109.1 (62.8–208.0)	100.2 (49.8–165.1)	0.515	0.13 (−0.26–0.53)
High frequency (HF), ms^2^	80.4 (36.0–165.8)	87.3 (44.0–180.6)	68.9 (30.8–163.4)	0.411	0.16 (−0.23–0.56)
LF/HF ratio, %	1.3 (0.7–2.7)	1.2 (0.6–2.4)	1.4 (0.7–2.9)	0.547	0.12 (−0.25–0.54)

WCI: without cognitive impairment; CIND: cognitively impaired and not demented; 1Q: first quartile; 3Q: third quartile; CI: confidence interval; SDNN: standard deviation of normal-to-normal intervals; rMSSD: root mean square of successive R–R interval differences; pNN50: percentage of successive R–R intervals that differ by more than 50 milliseconds (ms); LF: low frequency; HF: high frequency.

## Data Availability

The original contributions presented in this study are included in the article. Further inquiries can be directed to the corresponding author.

## Abbreviations

The following abbreviations are used in this manuscript:

1Q	first quartile
3Q	third quartile
ADLs	activities of daily living
ANCOVA	analysis of covariance
ANS	autonomic nervous system
BEI	baroreflex entropy index
BMI	body mass index
BP	blood pressure
CI	confidence interval
CIND	cognitively impaired and not demented
DBP	diastolic blood pressure
EBSERH	Brazilian Hospital Services Company
ECG	electrocardiogram
HF	high frequency
HRV	heart rate variability
HRVB	heart rate variability biofeedback
HU	Dr. Washington Antônio de Barros Teaching Hospital
IADLs	instrumental activities of daily living
ICF	informed consent form
LF	low frequency
MCI	mild cognitive impairment
MMSE	mini-mental state examination
η^2^_p_	partial eta squared
nVNS	non-invasive vagus nerve stimulation
OH	orthostatic hypotension
pNN50	percentage of successive R–R intervals that differ by more than 50 milliseconds
rMSSD	root mean square of successive R–R interval differences
SAH	systemic arterial hypertension
SBP	systolic blood pressure
SDNN	standard deviation of normal-to-normal intervals
T2DM	type 2 diabetes mellitus
TBM	total body mass
TMT-A	trail making test part A
TMT-B	trail making test part B
UNIVASF	Federal University of Vale do São Francisco
WCI	without cognitive impairment
